# Converging technologies: vagus nerve stimulation and brain-computer interfaces as catalysts for advancing post-stroke aphasia rehabilitation

**DOI:** 10.1097/JS9.0000000000003148

**Published:** 2025-08-06

**Authors:** Kaini Zhang, Gengbin Chen, Seong Hee Choi

**Affiliations:** a Department of Audiology and Speech-Language Pathology, Daegu Catholic University, Gyeongsan, Republic of Korea; b Postgraduate Research Institute, Guangzhou Sport University, Guangzhou, China

*Dear Editor,*

The recent thorough study of invasive and non-invasive vagus nerve stimulation (VNS) for stroke rehabilitation published in your journal, “Invasive or non-invasive vagus nerve stimulation modulation of brain function and remodeling after stroke: a review[1]” caught our attention, as well as the perceptive investigation of brain-computer interfaces (BCIs) for neurological disorders. “Brain–computer interfaces: the innovative key to unlocking neurological conditions[2].” These articles offer insightful summaries of quickly developing subjects. To broaden the conversation, we would like to examine a crucial but little-studied intersection: the complementary potential of VNS and BCIs, enhanced by artificial intelligence (AI) and digital health platforms, for improving language recovery in post-stroke aphasia. Post-stroke aphasia hinders effective communication and reduces the quality of the patient’s daily life.

To create a more conducive brain environment for recovery, the VNS review persuasively describes how VNS increases neuroplasticity, lowers inflammation, and encourages vascular regeneration. At the same time, the BCI review emphasizes how BCIs can decode brain signals related to language attempts and offer tailored feedback or help with communication. We suggest a revolutionary approach to aphasia through the integration of various modalities. Before or during BCI-guided language rehabilitation sessions, VNS may “prime” language networks, increasing plasticity. This could speed up learning and improve results beyond what either modality could achieve on its own. In this regard, non-invasive transcutaneous auricular VNS (taVNS) offers a particularly viable companion for non-invasive EEG-based BCIs[1].

Artificial intelligence serves as the cornerstone enabling this technological convergence. During combined VNS-BCI therapy, machine learning algorithms process multimodal neural (EEG/fNIRS) and physiological data streams to achieve three synergistic objectives: Firstly, they dynamically optimize VNS parameters (timing, intensity) and BCI feedback by adapting to real-time neural states and individual response patterns, overcoming limitations of fixed protocols noted in prior research^[1,2]^. Secondly, advanced neural decoders trained on extensive datasets enable accurate interpretation of complex communicative intent—including attempted speech and word retrieval—even under suboptimal signal conditions, significantly enhancing therapeutic feedback precision[3]. Thirdly, integrated smartphone/tablet applications coupled with wearable taVNS and dry-electrode EEG systems facilitate intensive, personalized rehabilitation extending beyond clinical settings, addressing the critical need for accessible long-term recovery solutions[4].

Closed-loop systems activating VNS *precisely* when BCIs detect engagement in language processing constitute a transformative frontier. This biomimetic approach could maximize neuromodulatory effects during neuroplastic windows[3]. Complementary digital platforms could track progress, adapt therapeutic difficulty, and deliver engaging exercises to enhance adherence

Although both reviews acknowledge the importance of future technology integration, more attention should be paid to its application in aphasia rehabilitation, as well as to the role of AI-driven personalization and closed-loop paradigms. By leveraging converging technologies between neuromodulation (VNS) and neural interface systems (BCI), driven by AI and supplied through digital health frameworks, we can develop genuinely revolutionary, patient-specific therapies to recover language function following a stroke. (We ensure this article is compliant with the TITAN Guidelines[5].)

## Data Availability

Not applicable.

## References

[1] Zhang J Zhou F Roberts N. Invasive or non-invasive vagus nerve stimulation modulation of brain function and remodeling after stroke: a review. Int J Surg 2025;111:2148–61. 39715153 10.1097/JS9.0000000000002173

[2] Zhang H Jiao L Yang S. Brain-computer interfaces: the innovative key to unlocking neurological conditions. Int J Surg 2024;110:5745–62. 39166947 10.1097/JS9.0000000000002022 PMC11392146

[3] Anumanchipalli GK Chartier J Chang EF. Speech synthesis from neural decoding of spoken sentences. Nature 2019;568:493–98. 31019317 10.1038/s41586-019-1119-1 PMC9714519

[4] Mihajlovic V Grundlehner B Vullers R Penders J. Wearable, wireless EEG solutions in daily life applications: what are we missing? IEEE J Biomed Health Inform 2015;19:6–21. 25486653 10.1109/JBHI.2014.2328317

[5] Agha R Mathew G Rashid R. Transparency in the Reporting of Artificial intelligence – The TITAN Guideline. Prem J Sci 2025;10:100082.

